# Anatomic trajectory for iliac screw placement adapts better to the morphological features of the pelvis of each individual than the S2 alar iliac screw: a radiological study

**DOI:** 10.1007/s00701-023-05692-6

**Published:** 2023-07-17

**Authors:** Luis Álvarez Galovich, Julia Montoya Bordón, Irantzu Castelbon Blanco, Alejandro Peiro, Charles Louis Mengis, Ángel R. Piñera, Félix Tomé-Bermejo, Jesus Gallego

**Affiliations:** 1 Unidad de Patología de Columna, Hospital Universitario Fundación Jiménez Díaz, Avda. Reyes Católicos, 2, 28040 Madrid, Spain; 2 Servicio de Radiodiagnostico, Hospital Universitario Fundación Jiménez Díaz, Avda. Reyes Católicos, 2, 28040 Madrid, Spain; 3 Servicio de Patología de Columna, Instituto de Investigación Sanitaria Fundación Jiménez Díaz, Avda. Reyes Católicos, 28040 Madrid, Spain; 4 Universidad Autónoma de Madrid, Faculty of Medicine and Surgery, Ciudad Universitaria de Cantoblanco, 28049 Madrid, Spain

**Keywords:** Sacropelvic fixation, Iliac screw, S2AI technique, AI technique, Anatomic correlation, Spine surgery

## Abstract

**Purpose:**

The iliac fixation (IF) through the S2 ala permits the minimization of implant prominence and tissue dissection. An alternative to this technique is the anatomic iliac screw fixation (AI), which considers the perpendicular axis to the narrowest width of the ileum and the width of the screw. The morphological accuracy of the iliac screw insertion of two low profile iliac fixation (IF) techniques is investigated in this study.

**Methods:**

Twenty-nine patients operated on via low profile IF technique were divided into two groups, those treated using 28 screws with the starting point at S2, and those treated with 30 AI entry point. Radiological parameters (Tsv-angle, Sag-Angle, Max-length, sacral-distance, iliac-width, S2-midline, skin-distance, iliac-wing, and PSIS distance) and clinical outcomes (early and clinic complications) were evaluated by two blinded expert radiologists, and the results were compared in both groups with the real trajectory of the screws placed.

**Results:**

Differences between ideal and real trajectories were observed in 6 of the 9 evaluated parameters in the S2AI group. In the AI group, these trajectories were similar, except for TSV-Angle, Max-length, Iliac-width, and distance to iliac-wing parameters. Moreover, compared with S2AI, AI provided better adaptation to the pelvic morphology in all parameters, except for sagittal plane angulation, skin distance, and iliac width.

**Conclusions:**

AI ensures the advantages of low profile pelvic fixation like S2AI, with a starting point in line with S1 pedicle anchors and low implant prominence, and moreover adapts better to the morphological features of the pelvis of each individual.

## Introduction

Pelvic fixation remains a challenging and controversial area in spine surgery. Anatomy, bone quality, and biomechanical forces are some reasons why surgeons continue to explore and study options for fixation in spinal deformity patients. Pelvic fixation is often used to correct pelvic deformity and is in widespread practice to reduce implant failure in long lumbar fusions to the sacrum and increase successful arthrodesis in the region. A variety of spinopelvic fixation techniques exist and have been described in the literature. Iliac screws, the Galveston technique, S2 pedicle screws, alar screws, and S2 alar screws have been reported to be effective in achieving rigid lumbosacral fixation. Among them, iliac screw fixation, with screws placed into the iliac wing from the posterior superior iliac spine (PSIS), has demonstrated to be the most effective method of sacropelvic fixation [5]. Long anchors projecting into the ilium provide optimal pelvic fixation, and, from the biomechanical point of view, iliac screws have advantages in axial compression, torsion, and pullout strength [3, 23]. However, the implant prominence over the PSIS may be problematic and the placement of iliac screws often requires muscle and adjacent skin dissection, additional connectors, or rod bending [7, 15]. Furthermore, the integrity of the placed iliac screws could be compromised by the harvesting of iliac crest bone graft, due to its proximity to the implant placement [10]. A previous method of iliac fixation, sacral alar iliac pelvic fixation (SAI), described in the literature [19], used screws originated in the sacral area that allowed the choice of starting point and trajectory. The iliac fixation through the S2 ala provides a starting point in line with S1 pedicle anchors while implant prominence and tissue dissection are minimized. This technique consists of finding a pathway from the S2 sacrum towards the ilium with fluoroscopic guidance, which results in less blood loss, lower infection rates, and more rapid postoperative recovery [12]. Sometimes, the pelvic morphology varies between individuals, and transfixation of the sacroiliac joint is not always possible. In the adult population, we commonly use two low profile techniques, the S2AI [18] and the AI, where the iliac screw entry point is located along the medial border of the PSIS of the iliac crest at its junction with the sacrum [6]_._

The purpose of this study was to assess the anatomic correlation between ideal and real trajectories of the screws, in a group of patients who underwent sacropelvic fixation with S2AI and with AI techniques, and to establish which one adapts better to the morphological features of any given pelvis.

## Material and methods

We retrospectively evaluated the radiographs of 29 patients who underwent spinal fusion with pelvic fixation between 2017 and 2019. The patients were divided into two groups: one group treated with 28 screws with the starting point at S2 (S2AI group), and the second one, treated with 30 screws with an entry point at AI (AI group).

The ideal trajectory, considering the width of the screw through the narrowest width of the ilium, was obtained by 2 blinded expert radiologists using a three-dimensional CT program (Alma). The results were matched with the real trajectory achieved by the implanted screws in both groups. The length and width of the screws, as well as any complications, were also recorded.

The present study was approved by the Clinical Research Ethics Committee of the Hospital.

### Surgical techniques

The S2AI and the AI techniques evolved out of the established lumbosacral and sacropelvic surgical techniques.

In the S2AI technique, the starting point for the sacral alar-iliac screw is located in the sacrum at the level of the lateral sacral crest, between the S1 and S2 foramen, angled 40° laterally and 40° caudally. The ideal trajectory of a sacral alar-iliac screw passes immediately above the sciatic notch, so that the bottom threads of the screw would be in contact with the cortical bone forming the upper limit of the notch, providing optimal pull-out strength [18].

**Fig. 1:**
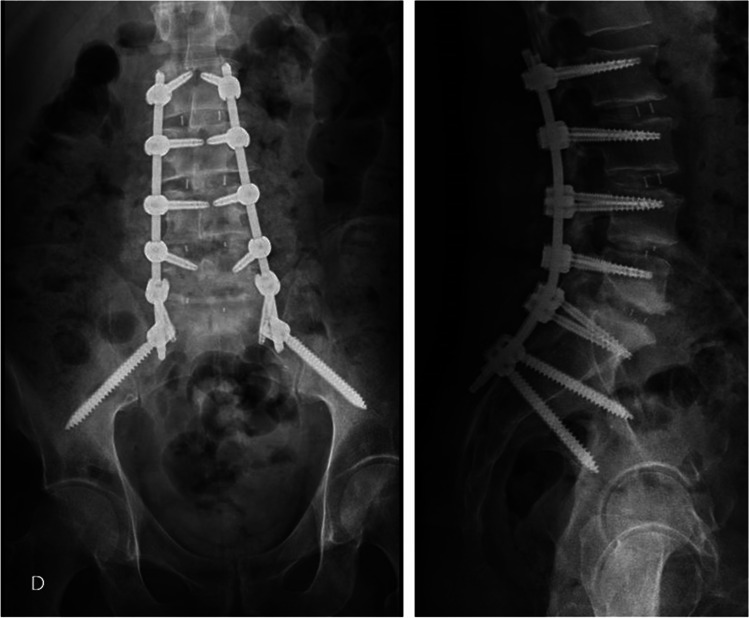
Lumbosacral construction with extension to the pelvis using AI screws. Note the AI screw head in line with the S1 screw (AP Projection) allowing a direct connection to the longitudinal rod through a gentle lordotic shaping of the rod (Lateral Projection) and without the need for connectors

AI technique was referred in 2009 as the “modified approach” of iliac bolt placement [6]. In the AI technique, the entry point is located directly in the ilium, along the medial aspect of the inner table of the ilium at its junction with the sacrum. The screw trajectory is 20° lateral and 30 to 35° caudal toward the anterior superior iliac spine. The goal is to get a screw passage within 1.5 to 2 cm above the sciatic notch. There is no risk of implant prominence since the head of the iliac screw is left deep and flush within the iliac bone. The head of the AI screw is placed a little more lateral than the S2AI screw, but still puts the screw directly in line with the S1 screw and, therefore, makes a direct linkage to the longitudinal rod through a gentle lordotic molding of the bar without the need for the use of connectors (Fig. 1).

Another advantage of the AI technique is that the screw is not placed through the SI joint into the ilium as is the case with the S2AI screw. From an entry point directly into the ilium, a gently advancing probe should allow the trajectory to remain between the internal and external tables of the ilium for the entire trajectory, preserving the SI joint.

### CT reconstruction and radiographic parameters

Dual-energy 128 × 2 multidetector CT scanner (Somatom Definition Flash, Siemens Medical Systems, Germany) was used to scan the lumbar spine with a layer thickness of 1.0 mm, dual voltage of 80 and 140 kV, current of 203 and 80 mAs, Caredose 40, and acquisition of 32 × 0.6 mm. The reconstruction was performed with a pitch of 0.6 mm and threshold of bone 50f. We reconstructed multiplanar images from axial scans at a distant workstation, Syngovia Siemens, with CT dual-energy work-flow.

**Fig. 2:**
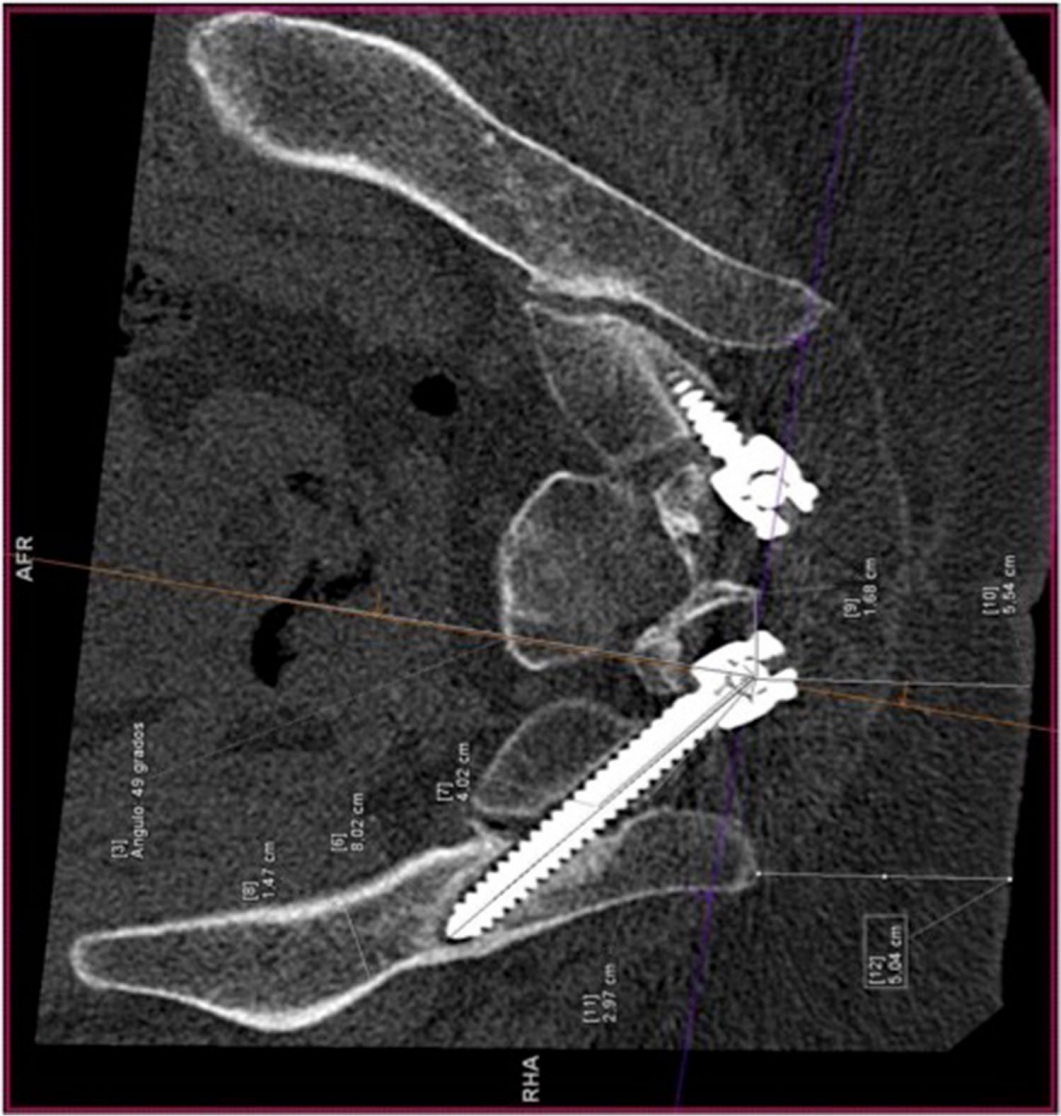
Ideal trajectory. CT imaging plane (projection of the line between PSIS and the anterior inferior iliac spine (AIIS)). The ideal trajectory is the line drawn perpendicular to the narrowest region

CT was rotated, until the screw holes on each side were observed in order to measure the different radiological parameters. To obtain the ideal trajectory on each patient [11], CT imaging planes were rotated until the viewing angle was perpendicular to the wing of the ilium. In this position, the projection of the line between the PSIS and the anterior inferior iliac spine (AIIS) is the longest and most horizontal, hence matching the greatest length and width of osseous channel. We draw a line perpendicular to the narrowest region, and the ideal entry point was obtained (Fig. 2).

Then the measurements were determined as follows [19]:

1. Sag angle: in the sagittal plane, defined as the angulation in the caudal trajectory.
2. Tsv angle: in the transverse plane, defined as the angulation in the lateral trajectory.
3. Max-length: defined as the maximal distance in a trajectory from S2 ala to the AIIS.
4. Sacral distance: defined as intrasacral trajectory length.
5. Iliac width: in the transverse plane, defined as the narrowest iliac width measured between the inner cortices.
6. S2 midline: defined as the distance of the starting point lateral from the middle line of S2.
7. Skin distance: defined as the distance of the starting point lateral from the skin.
8. Iliac wing: defined as the distance of the starting point lateral from the nearest iliac wing.

### Statistical analysis

Descriptive statistical analyses, including means, standard deviations, and percentages, were used to describe all parameters determined and to summarize radiographic results. The results were expressed as mean ± standard deviation. All statistical analyses were performed at the 0.05 level of global significance using two-sided tests. Testing for baseline differences between both groups was performed using Student’s test or Wilcoxon’s test for qualitative parameters and Chi- square test for qualitative parameters. Results were analyzed using IBM SPSS software version 21.0 (SPSS Inc., Chicago, IL, USA).

## Results

A total of 28 screws with the starting point at S2 and 30 screws with an entry point through the medial ilium cortical wall were analyzed from a total of 29 patients who underwent low profile pelvic fixation.

In the S2 group, the mean age and BMI were 71.75 years (range, 69–75), 27.49 kg/m^2^ (CI, 26–29), and the female/male ratio was 12/2; while in the AI group, they were 65.57 years (range, 62–69), 31.67 kg/m^2^ (CI, 30–34), and 10/5. The distribution of etiology was as follows: degenerative, 28.6%; deformity, 64.3%; fracture, 7.1% in the S2 group; and degenerative, 64.3%; deformity, 35.7% in the AI group (Table 1).

**Table 1:** Patients’ general characteristics

Variable	Total	S2	AI	*p*
Mean age, years (range)	65.20 (61–69)	71.75 (69–75)	65.57 (62–69)	*p* = 0.010*
BMI (confidence interval)	28.29 (26–31)	27.49 (26–29)	31.67 (30–34)	*p* = 0.327
Gender number (%):				*p* = 0.091
Female Male	44 (75.9%) 14 (24.1%)	24 (86%) 4 (14%)	20 (66.7%) 10 (33.3%)	
Etiology:				*p* = 0.043*
Degenerative Deformity Fracture Other	26 (46.4%) 28 (50%) 2 (3.6%) 0 (0%)	8 (28.6%) 18 (64.3%) 2 (7.1%) 0 (0%)	18 (64.3%) 10 (35.7%) 0 (0%) 0 (0%)	

^*^Statistical significance *p* < 0.05

In the AI group, the real trajectory described correlates statistically better with the ideal entry point described in most parameters, compared to the real and ideal trajectories for S2AI. While S2AI trajectories show differences in 6 out of the 9 evaluated parameters, real and ideal trajectories in AI were all similar, except for Tsv angle, Max-length, and iliac width (Table 2).

**Table 2:** The real and ideal trajectories for the S2AI and the AI groups

	S2			AI		
	Ideal	Real	*p*	Ideal	Real	*p*
Sag angle	31.4 ± 5.0	31.5 ± 5.7	0.929	31.3 ± 6.7	31.7 ± 6.2	0.841
Tsv angle	31.1 ± 3.0	43.4 ± 6.2	2.40E − 10**	31.5 ± 4.4	37.9 ± 10.5	0.003**
Max-length	132.4 ± 1.2	110.3 ± 1.7	3.12E − 08**	131.7 ± 1.2	120.8 ± 1.1	0.02*
Sacral distance	3.1 ± 1	26.2 ± 3.8	5.23E − 16**	3.4 ± 1.8	0.0 ± 0.0	0.08
Iliac width	15 ± 2.5	11.7 ± 3.3	1.00E − 07**	15.4 ± 1.6	13.0 ± 0.5	0.03*
S2 midline	37.6 ± 6.5	28.7 ± 1.8	8.30E − 08**	37.9 ± 6.7	37.8 ± 4.2	0.944
Skin distance	41.6 ± 11.9	45.5 ± 11.3	0.202	41.3 ± 8.9	44.1 ± 8.5	0.094
Iliac wing	0.25 ± 0.26	10.3 ± 0.63	1.00E − 06**	0.23 ± 0.23	0.1 ± 0.19	0.084

^*^Statistical significance *p* < 0.05

^**^Statistical significance *p* < 0.01

**Fig. 3:**
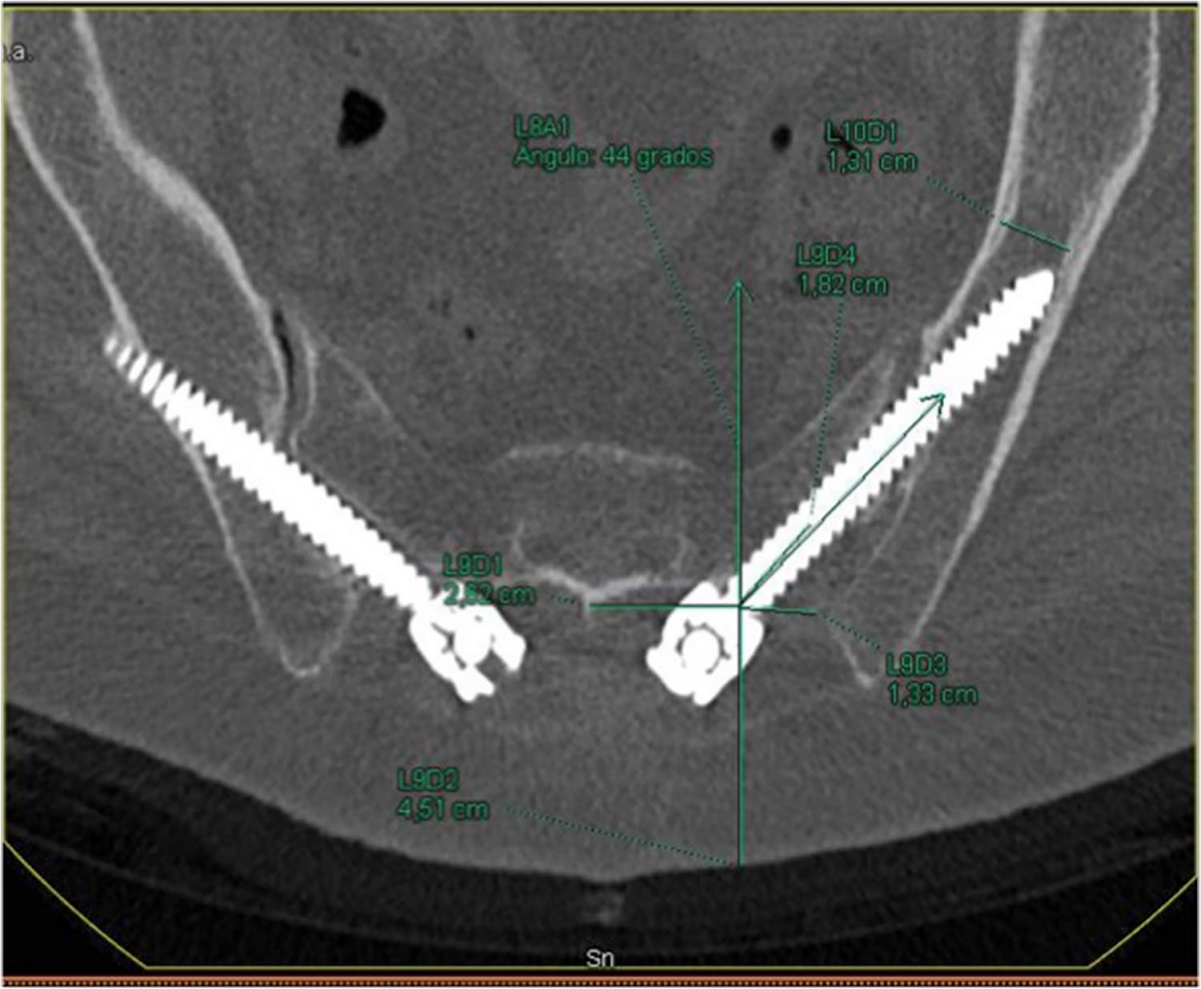
Bilateral S2AI screws. The right screw is not well oriented and breaks the cortical wall. On the left side, even though it is correctly oriented, it cannot go through the iliac isthmus

In order to determine which technique correlates better with the ideal trajectories, the percentage of difference between ideal and real trajectories for each technique was calculated parameter by parameter, awarding 100% to the ideal trajectory. Hereafter, differences between groups were analyzed by comparing the mean and the standard deviations of each parameter. As shown in Table 3, the AI technique adapts better to the morphological features of the pelvic in all parameters, except for the sagittal plane angulation (*p* = 0.832), skin distance (*p* = 0.497), and iliac width (*p* = 0.233), where no statistical differences were found between both techniques (Fig. 3).

**Table 3:** The morphological features of the pelvic in all parameters

	S2	AI	*p*
Sag angle	101.6 ± 19.6	102.6 ± 13.7	0.832
Tsv angle	140.9 ± 23.2	120.8 ± 33.3	0.010*
Max-length	84.1 ± 10.7	94.9 ± 4.6	4390E − 06**
Iliac width	77.2 ± 16.5	86.1 ± 35.5	0.233
S2 midline	78.2 ± 12.8	102.6 ± 21.1	2.09E − 06**
Skin distance	110.6 ± 4.6	109.8 ± 4.2	0.497
Iliac wing	76.1 ± 13.1	103.1 ± 19.9	2.18E − 06**

^*^Statistical significance *p* < 0.05

^**^ Statistical significance *p* < 0.01

**Fig. 4:**
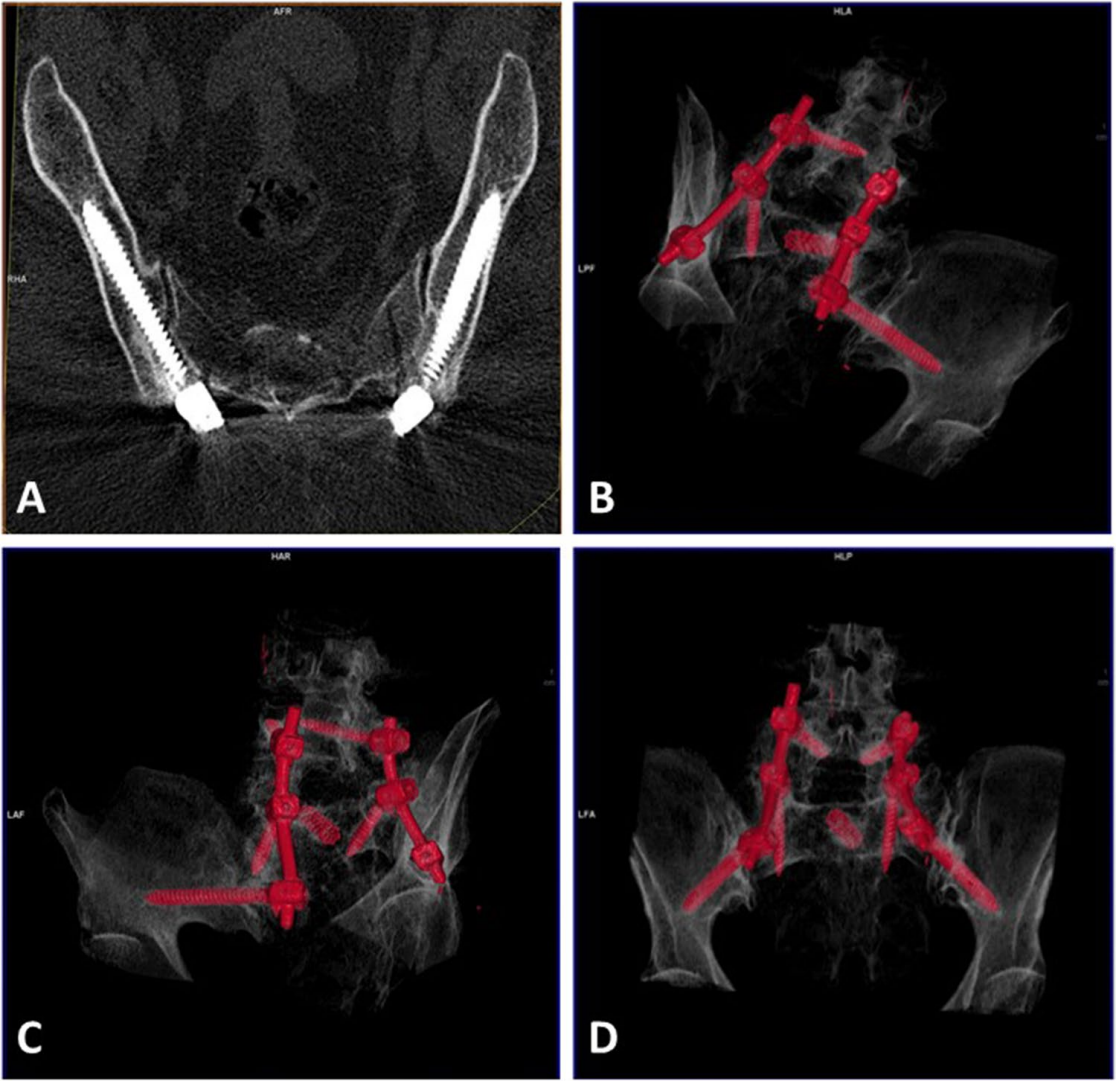
**A** An AI case, where iliac screws correlate with the ideal trajectory. On 3D reconstruction (**B**–**D**), on the inlet view of the pelvis, the screws are centered in the iliac view

In terms of complications, there were no cases of vascular or neurologic complications, implant prominence, anchor migration, pseudarthrosis, or iliac pain. Ten cases of external cortical wall breakage were reported in S2AI group (Fig. 4), whereas only 4 were observed in the AI group (*p* = 0.047). There were no cases of medial cortical wall breakage or radiculopathy in S2AI group compared with 1 instance each found in the AI group (one patient with a lumbar construction extended to the pelvis with postoperative radiculalgia unrelated to the iliac screw that improved after a short course of oral steroids).

## Discussion

Pelvic fixation has long been a challenging area in spinal surgery and multiple studies have shown a high rate of pseudarthrosis together with poor outcomes associated with fixation failure when screws in S1 promontory are implanted without supplemental fixation [8].

Recommended techniques for achieving additional fixation are the addition of an anterior interbody fusion device and performing supplemental fixation as a part of a complete lumbosacral fixation. This lumbosacral fixation includes alar screws, transiliac rods, and iliac post bolts. Iliac post bolts have been shown in biomechanical studies to be superior to other supplemental fixation techniques [21].

Traditional iliac post bolts may require an additional incision or a separated fascial incision, with dissection of the iliac crest and detachment of the muscles. It may also require the implantation of offset connectors. Furthermore, this technique may be not feasible in patients who previously underwent an iliac crest autograft harvest.

Low profile approaches were designed to address these problems. The entry points are in the sacrum, or in the inner table of the iliac crest, avoiding the need of additional incisions, or soft tissue dissections. Besides, the instrumentation is in-line with S1 screws, and is deep enough to avoid skin complications.

The S2AI has demonstrated a drastic reduction of complications compared with the traditional iliac screws in terms of infection rates or unplanned revision surgeries [13].

Based on our experience, we found more technical difficulties performing a S2AI technique than implementing an AI technique. Moreover, pelvic differences among sexes and regions, as previously described [2], add some technical difficulties to the S2AI, making its choice impossible in some cases.

Different anatomical analyses have demonstrated that the screw path from the PSIS towards AIIS provides the longest and thickest iliac plate anchor site [11].

In addition, AI adapts better to the morphological features of the patients and S2AI, as reported in the literature, has a high incidence of implant failure, probably caused by stresses on the interface screw head and screw shaft, so AI can be an effective alternative for sacropelvic fixation [4].

Our analysis illustrated that AI correlates better with the ideal trajectory (Fig. 4). In fact, the trajectory never crosses the sacroiliac joint and the entry point is similar to the one achieved by the longest and thickest screw possible that could fit into the channel. The narrowest point of the iliac channel is just above the sciatic notch, where the anchoring for iliac screws is critical to avoid violations toward the sciatic notch [22]. The feasibility of S2AI screw insertion, into the sacrum and ilium, has been identified and previously described for clinical practice [9]. With screws similar to those used in our series, increased feasibility could be achieved given that it would be easier to get its modification once the SI joint is crossed.

From our data, we can understand that AI and S2AI screws have the same advantages in terms of avoiding implant prominence. Both techniques get the same skin distance, which is bigger than the one expected in an ideal trajectory.

The insertion maneuvers may be performed with fluoroscopy or computer-assisted navigation. The radiology has already been described [16], and it is especially recommended for the S2AI technique [17]. With the exception of the first S2AI case (with operative time extension), we have performed all the insertions without using fluoroscopy or computer-assisted navigation. That could explain the large number of outer iliac table penetrations, especially in the S2AI group, where the direction of the screw is fixed once it crosses the SI joint, being difficult its modification once into the channel. We can conclude that fluoroscopy is mandatory to place S2AI screws, while it may not be necessary for the anatomical approach. In our series, the number of penetrations of the iliac table after performing an AI approach was similar to other series with S2AI approach [1]. However, these penetrations were much higher in the S2AI technique, using fluoroscopy, where a rate of 15% has been described [14]. Despite increasing experience in the use of CT-guided navigation for pedicle screw placement, there is still limited but promising knowledge examining the use of intraoperative CT guidance for pelvic screw placement. Potential benefits of CT navigation for pelvic screw placement may include greater precision and larger, longer screws [20].

The purpose of the present study is to investigate the morphologic accuracy of iliac screw insertion using two different profile techniques. S2AI has demonstrated feasibility, efficiency, and clinical safety. It is probably the best option in those cases with a previous iliac crest harvesting due to its anchoring in the anterior position of the ilium. However, AI has the advantages of the S2AI, and moreover fits better with the ideal trajectory of an iliac screw, although more studies are required in order to verify if pseudarthrosis and clinical or biomechanical results could change. The diverse morphology found in any patient made us recommend a preoperative CT study for each case in order to decide the best choice.

## Conclusion

The anatomic entry point has the advantages of low profile pelvic fixation, with a starting point in line with S1 pedicle anchors and low implant prominence, but, moreover, it adapts better to the morphological features of the pelvis of each individual and reduces the risk of external wall breakage.
